# Cardioprotective effects of Notoginsenoside R1 against ischemia/reperfusion injuries by regulating oxidative stress- and endoplasmic reticulum stress- related signaling pathways

**DOI:** 10.1038/srep21730

**Published:** 2016-02-18

**Authors:** Yingli Yu, Guibo Sun, Yun Luo, Min Wang, Rongchang Chen, Jingyi Zhang, Qidi Ai, Na Xing, Xiaobo Sun

**Affiliations:** 1 Beijing Key Laboratory of Innovative Drug Discovery of Traditional Chinese Medicine (Natural Medicine) and Translational Medicine, Institute of Medicinal Plant Development, Peking Union Medical College and Chinese Academy of Medical Sciences, Beijing, 100193, China; 2 Key Laboratory of Bioactive Substances and Resource Utilization of Chinese Herbal Medicine, Ministry of Education, China; 3 Zhongguancun Open Laboratory of the Research and Development of Natural Medicine and Health Products, China; 4 Key Laboratory of Chinese Materia Medica, Heilongjiang University of Chinese Medicine, Harbin, 150040, China

## Abstract

*Background*: Recent reports suggested the involvement of oxidative stress- and endoplasmic reticulum stress (ERS)-associated pathways in the progression of ischemia/reperfusion (I/R) injury. Notoginsenoside R1 (NGR1) is a novel saponin isolated from *P. notoginseng*, which has a history of prevention and treatment of cardiovascular diseases. *Objective*: We aimed to examine the cardioprotective effects of NGR1 on I/R-induced heart dysfunction *ex vivo* and *in vitro. Methods*: H9c2 cadiomyocytes were incubated with NGR1 for 24 h and exposed to hypoxia/reoxygenation. Isolated rat hearts were perfused by NGR1 for 15 min and then subjected to global ischemia/reperfusion. Hemodynamic parameters were monitored as left ventricular systolic pressure (LVSP), heart rate, and maximal rate of increase and decrease of left ventricular pressure (±dP/dt max/min). *Results*: NGR1 pretreatment prevents cell apoptosis and delays the onset of ERS by decreasing the protein expression levels of ERS-responsive proteins GRP78, P-PERK, ATF6, IRE, and inhibiting the expression of pro-apoptosis proteins CHOP, Caspase-12, and P-JNK. Besides, NGR1 scavenges free radical, and increases the activity of antioxidase. NGR1 inhibits Tunicamycin-induced cell death and cardic dysfunction. *Conclusion*: We elucidated the significant cardioprotective effects of NGR1 against I/R injuries, and demonstrated the involvement of oxidative stress and ERS in the protective effects of NGR1.

Ischemic heart disease is one of the most severe cardiovascular diseases, and it represents a major contributor to morbidity and mortality worldwide^1^. After the great success of therapies to reduce ischemic injury, the scientific community’s attention has been focused on reducing ischemia/reperfusion (I/R) injury^2^,^3^,^4^, which currently lacks an effective clinical therapy. Typically, myocardial I/R injury cause irreversible cell apoptosis, necrosis, and cardiomyocyte death, which can lead to arrhythmias, microvascular dysfunction, myocardial stunning and heart failure^5^.

Endoplasmic reticulum stress (ERS) has recently attracted significant attention, and ERS-initiated apoptotic signalling has been implicated in I/R myocardium^6^,^7^. Both the depletion of the oxygen/glucose supply and the sudden increase in oxygen free radicals potentially trigger oxidative stress^8^, with the production of nitric oxide (NO) and other reactive oxygen species (ROS). These disturbances in cellular redox regulation interfere with the function of disulphide bonding in the lumen of the ER, leading to the unfolded protein response (UPR)^9^,^10^. The UPR mediates ERS through three ER transmembrane receptors: pancreatic ER kinase -like or PKR-like ER kinase (PERK), activating transcription factor 6 (ATF6) and inositol-requiring enzyme-1α (IRE1)^9^,^11^,^12^. Recent reports have suggested that the UPR signalling switches from pro-survival (adaptive response) to pro-apoptosis (maladaptive response) through the transcriptional induction of C/EBP homologous protein (CHOP), the activation of c-Jun N-terminal kinase (JNK) and Caspase-12-dependent pathways^13^,^14^,^15^.

*Panax notoginseng* (Burk.) F. H. Chen is an ancient medicinal plant in China that was first recorded in the Compendium of Materia Medica by Shi-zhen Li in 1590; it is known for its beneficial effects on the prevention and treatment of cardiovascular and cerebral vascular diseases^16^,^17^. In clinical settings, *P.notoginseng* is mainly used for analgesia and haemostasis (Chinese Pharmacopoeia, 2010). Notoginsenoside R1 (NGR1) (Fig. 1a) is a novel saponin that from *P. notoginseng*. Our previous studies have demonstrated that NGR1 provides neuroprotection in H_2_O_2_-induced oxidative damage in PC12 cells^18^. NGR1 also protects PC12 neuronal cells from Aβ 25–35-induced neurotoxicity by inhibiting the oxidative stress, apoptosis, and stress-activated MAPK signalling pathways^19^. Moreover, remarkable evidence has indicated NGR1’s protective roles, such as anti-oxidation, anti-inflammatory, anti-angiogenic, and anti-apoptosis^20^,^21^. However, whether NGR1 provides cardioprotection against I/R injury, or whether its protective effects are connected with the inhibition of oxidative stress-and ERS-associated apoptosis pathways remains unclear.

In this study, multiple approaches were employed to explore the cardiac protective effects and the underlying mechanisms of NGR1 against myocardial I/R injuries in cultured cardiomyocytes and in isolated rat hearts. Especially, we established ERS models by specific ERS inducer tunicamycin (TM). We found that oxidative stress and ERS induced by I/R were largely inhibited by NGR1. We elucidated the significant protective effects of NGR1 against I/R injuries in heart through the inhibition of oxidative stress- and ERS- associated apoptosis.

## Results

### Effects of reoxygenation time on cell viability and ERS signaling pathways in the H9c2 cardiomyocytes

**Figure 1:**
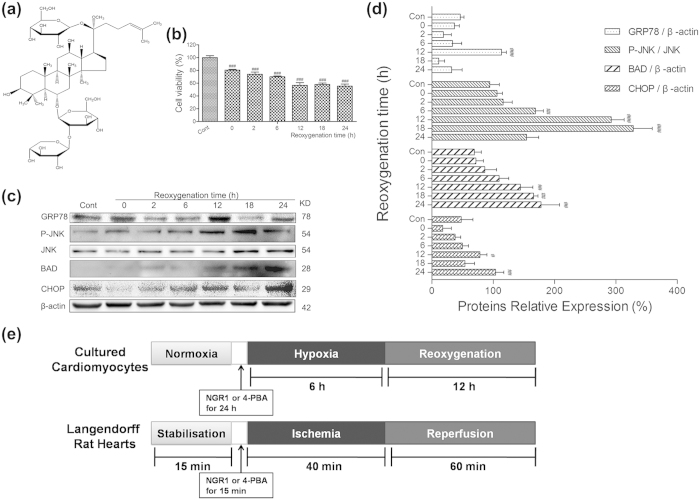
Effects of reoxygenation time on H9c2 cardiomyocytes cell viability and ERS signaling pathways, and the experimental design. H9c2 cardiomyocytes were subjected to 6 hours of hypoxia and then exposed to different duration (0, 2, 6, 12, 18, 24 h) of reoxygenation. The cell viability was detected using MTT assay, and the cell lyser was analysed by western blotting. (**a**) Chemical structure of NGR1; (**b**) Effects of reoxygenation time on H9c2 cardiomyocytes cell viability; (**c**) Immunoblot analysis of ERS-associated GRP78, P-JNK, JNK, BAD, CHOP, and β-actin were performed in cell lyser; (**d**) The relative protein expression of GRP78, BAD, CHOP to β-actin, and P-JNK to JNK are expressed in the bar graphs; (**e**) The experimental design of cultured cardiomyocytes and Langendorff-perfused rat hearts. ^#^*P* < 0.05 versus the control group, ^##^*P* < 0.01 versus the control group, ^###^*P* < 0.001 versus the control group.

H9c2 cardiomyocytes were exposed to hypoxia for 6 h, followed by reoxygenation for 24 h. Cell viability was detected at 0, 2, 6, 12, 18 and 24 h after reoxygenation by MTT assay. The percentage of cell viability in each group was calculated relative to control. As shown in Fig. 1b, 6 h of hypoxia caused a decrease in cell viability approximately by 19.6%, and reoxygenation provoked further decline in a time-dependent manner. However, the viability at 12 h after reoxygenation was around 56.7%, and decreased by a much lower speed after that. Besides, we examined the expression of proteins in ERS and its associated apoptosis signaling pathways at different times (0 h to 24 h), to elucidate the molecular mechanism of H/R-induced ERS in H9c2 cardiomyocytes. As shown in Fig. 1c, the expression level of GRP78 was markedly increased after 12 h of reoxygenation and decreased after that. The phosphorylation of JNK was quickly induced by reoxygenation and reached the peak at 18 h. And increased BAD and CHOP remained active until 24 h after reoxygenation (Fig. 1c,d, *P* < 0.01 or *P* < 0.005). These results indicate that ERS was induced and activated after 12 h of reoxygenation, and apoptosis was initiated soon after that. After 18 h of reoxygenation, ERS was weakened and apoptosis was reinforced. Based on the results, hypoxia for 6 h and reoxygenation for 12 h were selected as optimal conditions for the following experiments.

### NGR1 protects H9c2 cardiomyocytes from H/R-induced cell death, intracellular ROS accumulation, and mitochondrial membrane depolarization

**Figure 2:**
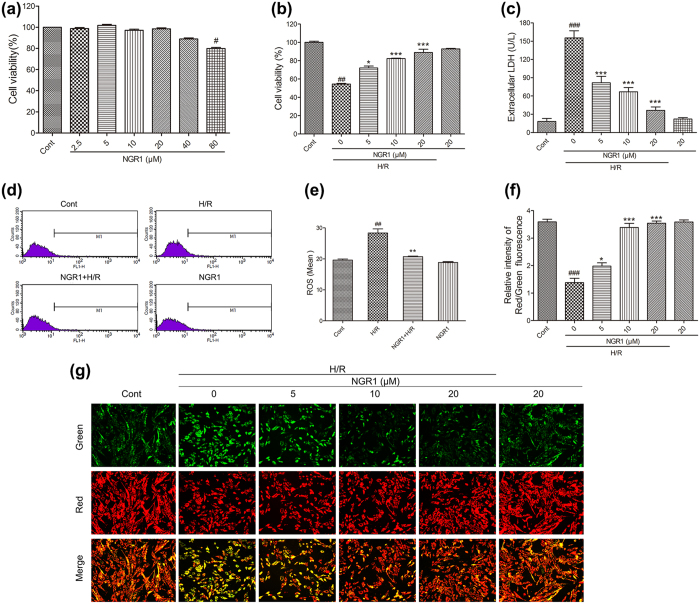
Effects of NGR1 on H/R-induced cell death, depolarization of mitochondrial membrane and intracellular ROS accumulation in H9c2 cardiomyocytes. H9c2 cardiomyocytes were incubated with indicated concentration of NGR1 (2.5, 5, 10, 20, 40 and 80 μM) for 24 h and then exposed to 6 h of hypoxia and 12 h of reoxygenation. (**a**) NGR1 had no toxic effect on cell viability with concentrations under 20 μM; Effects of NGR1 on H/R-induced cell viability were detected by (**b**) MTT and cell death by (**c**) extracellular LDH; (**d**) Intracellular ROS levels evaluated using a FACSCalibur flow cytometer; (**e**) Bar diagram showing intracellular ROS level in H9c2 cardiomyocytes; (**f**) Bar graphs and (**g**) representative images of JC-1 red/green cells and merges showed that NGR1 increased the ratio of red to green fluorescence intensity. ^#^*P* < 0.05 versus the control group, ^##^*P* < 0.01 versus the control group, ^###^*P* < 0.001 versus the control group, *P < 0.05 versus the H/R group, **P < 0.01 versus the H/R group, ***P < 0.001 versus the H/R group.

The potential cardioprotective effects of NGR1 on H9c2 cardiomyocytes against H/R injury were estimated using MTT and LDH assays. As shown in Fig. 2a, there was no significant difference in cell viability between the groups that underwent incubation for 24 h with low concentrations of NGR1 (5, 10, 20, and 40 μM) and the control group (*P* > 0.05), although high concentrations of NGR1 (80 μM) decreased cell viability (*P* < 0.05). Moreover, treatment of H9c2 cells with hypoxia for 6 h and reoxygenation for 12 h reduced the cell viability to approximately 50% that of the control group (Fig. 2b, *P* < 0.01), and NGR1 supressed this decrease in a concentration-dependent manner (5, 10, and 20 μM) (Fig. 2b, *P* < 0.05 or *P* < 0.01). 20 μM of NGR1 incubation increased the cell viability to approximately 89% that of the control group. LDH leakage, as a biomarker of cell death, was also detected. As shown in Fig. 2c, H/R treatment significantly increased the LDH leakage from 18 to 155 compared with the control group, and NGR1 preconditioning effectively decreased the LDH release (Fig. 2c, *P* < 0.01 or *P* < 0.001). NGR1 treatment alone (20 μM) had no effect on cell viability or LDH release (*P* > 0.05). Oxidative damage mediated by free radicals is also a contributing factor to H/R-induced injury in cardiomyocytes. The intracellular ROS level was assessed by measuring carboxy-H2DCFDA fluorescence. Compared with the control, H/R treatment increased the intracellular ROS level in H9c2 cells by almost 1.5-fold. However, NGR1 preconditioning significantly inhibited this increase (Fig. 2d,e, *P* < 0.01). The change in mitochondrial membrane potential was assessed using JC-1 staining. Treating the H9c2 cells with H/R resulted in a pronounced decrease in the ratio of red to green fluorescence intensity (Fig. 2f,g, *P* < 0.001), which is a sign of the early stages of cell apoptosis, and NGR1 preconditioning significantly inhibited this H/R-induced effect and increased the red to green ratio by a large margin (*P* < 0.05 or *P* < 0.001). The results indicated that preconditioning H9c2 cardiomyocytes with NGR1 was able to protect against cardiac injury induced by H/R.

### NGR1 protects against H/R-induced cell apoptosis in H9c2 cardiomyocytes

**Figure 3:**
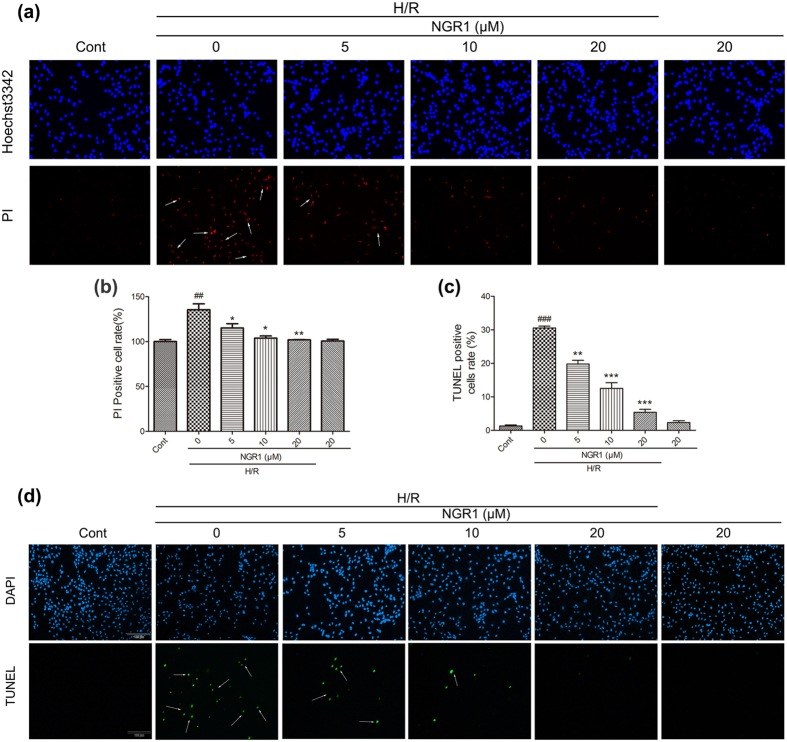
Myocardial apoptosis was determined by Hoest33342/PI double staining and TUNEL assay. (**a**) Representative images of Hoest33342 positive cells or PI positive or both positive cells; (**b**) Bar diagram showing quantitative data of PI-positive cell rate compared with the control group; (**c**) Representative images of TUNEL-positive nuclei in green fluorescent colour and total nuclei staining with 4,5-diamino-2-phenylindole (DAPI); (**d**) Bar diagram showing quantitative data of TUNEL positive nuclei in myocardium. ^##^*P* < 0.01 versus the control group, ^###^*P* < 0.001 versus the control group, *P < 0.05 versus the H/R group, **P < 0.01 versus the H/R group, ***P < 0.001 versus the H/R group.

The cardioprotective effects of NGR1 against H/R-induced apoptosis were further investigated using Hoest33342/PI double staining and TUNEL staining. As shown in Fig. 3a,b, the PI positive cell rate was substantially increased by H/R from 100 to 135.5 compared with the control (*P* < 0.01). However, different concentrations of NGR1 preconditioning significantly reversed the increase to 115 (5 μM), 103(10 μM) and 101 (20 μM), respectively (*P* < 0.05 or *P* < 0.01). Consistent with this, the TUNEL-positive cell (green) rate was increased to 30.6% by H/R compared with 1.3% in the control group. and NGR1 preconditioning significantly reduced the percentage to 19.8%, 12.5%, and 5.4%, respectively, in a concentration-dependent manner (Fig. 3c,d, corresponding to 5, 10, and 20 μM of NGR1, respectively, *P* < 0.01 or *P* < 0.001), whereas NGR1 treatment alone had no significant effect on DNA fragmentation (*P* > 0.05). These results indicated that NGR1 rescues H9c2 cardiomyocytes from H/R-induced apoptosis.

### NGR1 ameliorated I/R-induced heart dysfunction, myocardial cell degeneration, suppressed oxidative stress, and reduced the I/R-regulated overexpression of apoptosis-associated proteins in Langendorff-perfused rat hearts

**Figure 4:**
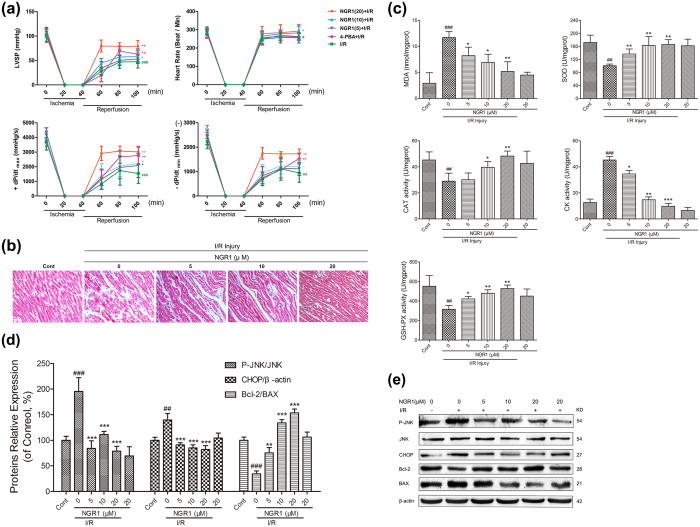
Effects of NGR1 on I/R-induced heart dysfunction, myocardial cell degeneration, imbalance in redox state, and activated apoptosis pathways in the isolated Langendorff-perfused rat hearts. After 15 min of NGR1 or 4-PBA processing, adult rat hearts were subjected to 40 min of global ischemia followed by 60 min of reperfusion. (**a**) NGR1 showed better effects on improving LVSP, heart rate, + dp/dt_max_, and -dp/dt _min_ compared with 4-PBA in the Langendorff I/R model; (**b**) Histopathological examination showed NGR1’s cardioprotection of the I/R-impaired hearts; (**c**) The intracellular antioxidant enzyme activity in the isolated rat hearts were examined by measuring MDA, SOD content, and CAT, CK, GSH-Px activities; (**d**,**e**) Immunoblot analysis of NGR1’s effects on I/R-induced relative overexpression of apoptosis-associated proteins: P-JNK to JNK, CHOP to β-actin, and Bcl-2 to BAX. ^#^*P* < 0.05 versus the control group, ^##^*P* < 0.01 versus the control group, ^###^*P* < 0.001 versus the control group, *P < 0.05 versus the I/R group, **P < 0.01 versus the I/R group, ***P < 0.001 versus the I/R group.

To determine the therapeutic implications of NGR1on I/R injuries, we employed an *ex vivo* Langendorff model.4-Phenylbutyric acid (4-PBA), which is an ERS inhibitor, was used as a positive control. As illustrated in Fig. 1e, after a 15-min stabilization period and 15 min of drug processing, adult rat hearts were subjected to 40 min of global ischemia followed by 60 min of reperfusion. The LVSP, heart rate and +dp/dt_max_ are important parameters of left ventricular systolic function, whereas −dp/dt_min_ represents left ventricular diastolic function. The results presented in Fig. 4a showed that I/R treatment significantly decreased the levels of heart LVSP, heart rate, the +dp/dt_max_, and −dp/dt_min_ to 43%, 89%, 41%, and 42%, respectively, compared with the levels obtained before I/R (the control group is not shown) (all *P* < 0.05). However, in the NGR1 + I/R treatment groups, the levels of LVSP, +dp/dt_max_, and the −dp/dt_min_ were significantly improved (all *P* < 0.05), although the heart rate exhibited no significant differences among the groups (all *P* > 0.05). In the NGR1 high concentration group (20 μM) in particular, the LVSP was increased to approximately 76% of that before I/R, and the +dp/dt_max_ and −dp/dt_min_ were increased to 78% and 71%, respectively, which are all much higher than those in the 4-PBA treatment group (all *P* < 0.05). Moreover, 4-PBA (3 mM) treatment also suppressed the I/R-induced decrease in heart LVSP, +dp/dt_max_, and −dp/dt_min_, although with a relatively slower speed (approximately 40 min after reperfusion) compared with the NGR1 + I/R treatment groups. Consistently, the results of the histopathological examination confirmed that NGR1 preconditioning significantly suppressed the myocardial cell degeneration, rupture, interstitial oedema, and inflammatory cell infiltration induced by I/R (Fig. 4b, *P* < 0.05). Taken together, these results suggest that NGR1 is capable of ameliorating I/R-induced heart dysfunction in the Langendorff-perfused rat hearts and showed better effects than the 4-PBA positive control group.

ROS are enzymatically processed by superoxide dismutase (SOD), catalase and glutathione peroxidase, the latter of which depends on adequate reserves of reduced glutathione^22^. The effects of I/R treatment on antioxidant enzyme activities were further evaluated. The results presented in Fig. 4c indicated that I/R caused significant decreases in SOD, CAT, and GSH-Px activities (Fig. 4c, *P* < 0.01) and increases in lipid peroxidation (MDA) and CK production. However, these changes were effectively suppressed by NGR1 preconditioning in a dose-dependent manner, which indicated that NGR1 protects the myocardium from I/R-induced oxidative stress and oxidative damage.

The potential cardioprotective effects of NGR1 against I/R-induced cell apoptosis were further explored by immunoblotting analysis. Total soluble protein of the Langendorff-perfused rat hearts was extracted and performed in the following experiments. It has been well established that C/EBP homologous protein (CHOP) is an important pro-apoptotic transcription factor during ER-initiated apoptosis; in addition, phosphorylated JNK and activated Caspase-12 are involved in this process^15^. As shown in Fig. 4d,e, I/R significantly increased the relative protein levels of pro-apoptotic proteins P-JNK and CHOP to 1.95-, 1.40- fold of that in the control group, which was associated with the downregulation of the anti-apoptotic protein Bcl-2 to 0.34- fold of the control group (*P* < 0.01). However, this processing was suppressed by NGR1 in a dose-dependent manner. Compared to the I/R group, NGR1 treatment significantly increased myocardium Bcl-2 protein expression to 0.75, 1.34, and 1.53, and decreased CHOP protein expression to 0.90, 0.85, and 0.82, P-JNK protein expression to 0.84, 1.11, and 0.78 (corresponding to 5, 10, and 20 μM of NGR1, respectively, Fig. 4d,e, *P* < 0.05, *P* < 0.01, or *P* < 0.001). In addition, NGR1 treatment alone had no significant effects on the expression levels of the proteins mentioned above (*P* > 0.05). These results suggested that NGR1 was capable of preventing the I/R-initiated overexpression of apoptosis-associated proteins.

### NGR1 suppressed I/R-induced UPR and ERS pathways thus alleviated ERS-associated apoptosis and provided cardioprotection

**Figure 5:**
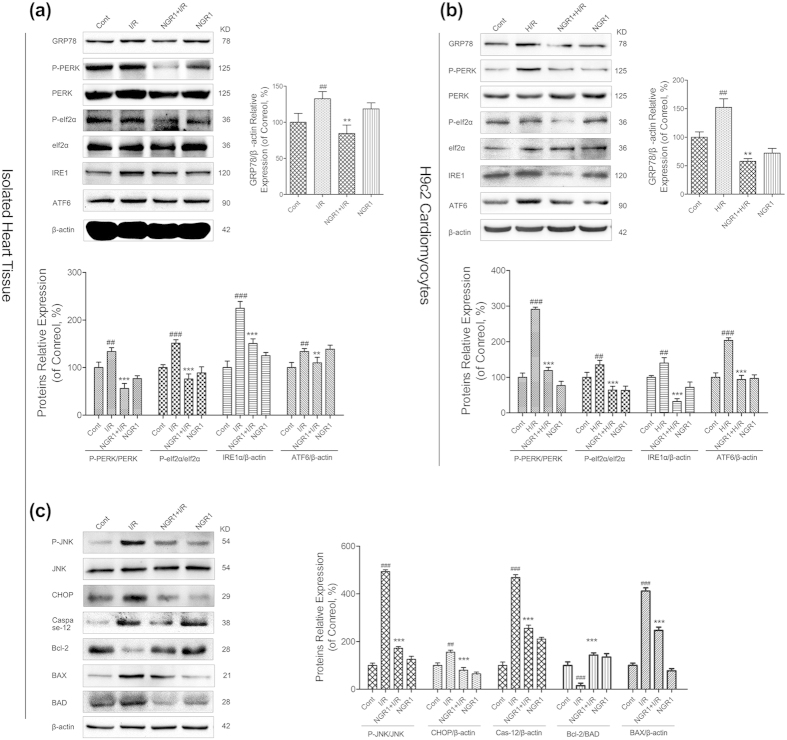
Effects of NGR1 and I/R on the expression of UPR pathways and ERS- associated apoptosis proteins. The expression levels of ERS and UPR pathway proteins in langendoff-perfused rat hearts (**a**) and in H9c2 cardiomyocytes (**b**) were detected using an immunoblotting assay. The relative protein expression of GRP78, IRE1, ATF6 to β-actin, and P-PERK to PERK, P- eIf2α to eIf2α are expressed in the bar graphs. Then we examined the ERS-associated apoptosis proteins (**c**) including P-JNK, JNK, CHOP, Caspase-12, Bcl-2, BAX, and BAD in the tissue of isolated rat hearts. The results are expressed as the mean ± SD from three independent experiments. ^#^*P* < 0.05 versus the control group, ^##^*P* < 0.01 versus the control group, ^###^*P* < 0.001 versus the control group, *P < 0.05 versus the I/R group, **P < 0.01 versus the I/R group, ***P < 0.001 versus the I/R group.

To confirm the involvement of ERS in the process of I/R-induced apoptosis and the role of ERS in NGR1’s cardioprotective effects, the ERS-responsive marker GRP78 and the ERS sensors ATF6, PERK, eIf2α and IRE1 were evaluated both in Langendorff-perfused rat hearts and in H9c2 cardiomyocytes. As shown in Fig. 5a,b, the expression level of GRP78 was increased to 1.32-fold in isolated rat hearts and 1.52-fold in H9c2 cells in the I/R-treated group compared with the control group (*P* < 0.01). And significant increases were also observed in the phosphorylation of PERK and eIf2α, and the protein expression of IRE1and ATF6 to varying degrees (1.34-, 1.52-, 2.25-, and 1.34-fold of the control in Langendorff-perfused rat hearts, and 2.90-, 1.35-, 1.40-, 2.03-fold of the control in H9c2 cells, respectively, *P* < 0.05 or *P* < 0.01). However, the groups that were preconditioned with NGR1 (20 μM) showed a significant reduction in the expression levels of GRP78 and the ERS sensors P-PERK, IRE1, and ATF6, compared with the I/R group (Fig. 5a,b, *P* < 0.01 or *P* < 0.001), both in Langendorff-perfused rat hearts and in H9c2 cardiomyocytes. In particular, P-PERK, and P-eIf2α were greatly inhibited by NGR1 (20 μM) to 42%, 50% of that in I/R group in isolated heart tissue (Fig. 5a, *P* < 0.01 or *P* < 0.001), and 41%, 47% of that in the H/R group in H9c2 cells (Fig. 5b, *P* < 0.01 or *P* < 0.001), respectively. Moreover, pharmacological intervention with NGR1 (20 μM) significantly suppressed the I/R-induced upregulation of the pro-apoptotic proteins P-JNK, CHOP, BAD, BAX, and Caspase-12 compared with the control (Fig. 5c, *P* < 0.01 or *P* < 0.001), increased the expression levels of the anti-apoptotic protein Bcl-2 and inhibited the apoptosis induced by I/R. Collectively, these results indicated that ERS was involved in the I/R-induced myocardium injury and mediated cardiomyocyte apoptosis. Most importantly, the ERS and its associated apoptosis were markedly suppressed by NGR1 preconditioning.

### NGR1 suppressed TM-induced myocardial injury both in Langendorff-perfused rat hearts and H9c2 cardiomyocytes

**Figure 6:**
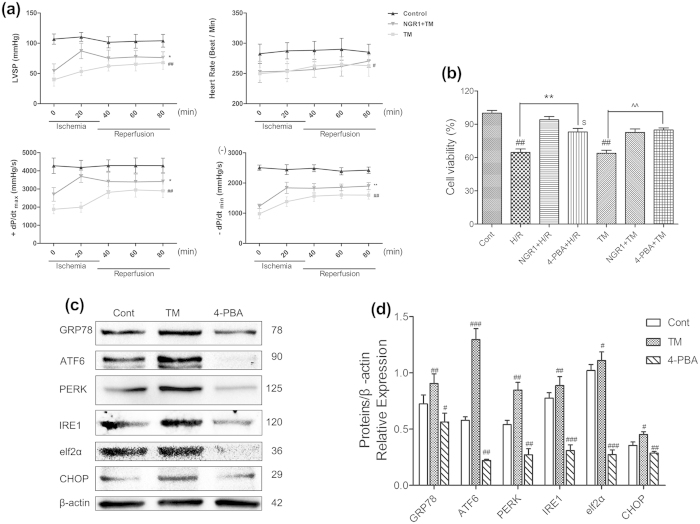
Effects of NGR1 on TM-induced myocardial injury in Langendorff- perfused rat hearts and H9c2 cardiomyocytes. The hearts with or without 15 min of NGR1 (20 μM) preconditioning were exposed to TM (3 μM) for 15 min, then the solution was replaced by normal perfusate and perfused for another 80 min, which was presented in Fig. 1(e). The normal group was perfused with normal perfusate for 110 min. (**a**) Effects of NGR1 preconditioning on TM-impaired cardio dysfunction in LVSP, heart rate, + dp/dt_max_, and −dp/dt_min_; (**b**) Effects of NGR1 preconditioning on cell viability of H9c2 cardiomyocytes exposed to I/R or TM; (**c**) TM treatment caused an upregulation of ERS associated proteins, whereas 4-PBA significantly suppressed them; (**d**) The relative protein expression of GRP78, ATF6, PERK, IRE1, eIf2α, and CHOP to β-actin were represented in bar graph. ^#^*P* < 0.05 versus the control group, ^##^*P* < 0.01 versus the control group, ^###^*P* < 0.001 versus the control group, *P < 0.05 versus the I/R group, **P < 0.01 versus the I/R group, ***P < 0.001 versus the I/R group, ^$^*P* < 0.05 versus the NGR1 + H/R group, ^^^^*P* < 0.01 versus the TM group.

To further investigate the mechanisms responsible for the protective effects of NGR1, we used tunicamycin (TM), which is a typical inducer of ERS, to initiate ERS and myocardium damage. 4-PBA was used as a positive control. We examined the function of NGR1 on TM-induced myocardial injury. As the results showed in Fig. 6, pretreatment with 3 μM of TM for 15 min significantly damaged the heart function of the Langendorff-perfused rat hearts (Fig. 6a, *P* < 0.05, or *P* < 0.01) and incubation with 3 μM of TM for 6 h decreased the cell viability of the H9c2 cardiomyocytes (Fig. 6b, *P* < 0.05, or *P* < 0.01). However, as shown in Fig. 6a, pre-treatment with NGR1 (20 μM) significantly improved the TM-impaired cardio dysfunction in LVSP, +dp/dt_max_, and – dP/dt_min_ (*P* < 0.05), although no significant differences were found in the heart rate (*P* > 0.05). Interestingly, NGR1 (20 μM) treatment showed better cardioprotective effects than 4-PBA on H/R-induced H9c2 cell death but did no better on improving the TM-induced cell death (Fig. 6b, *P* < 0.01). Moreover, we examined the SOD activity and MDA production of the isolated heart, and assessed the intracellular ROS levels in H9c2 cells. There was no significant difference found between the groups (Supplementary Figure 1, *P* > 0.05).

**Figure 7:**
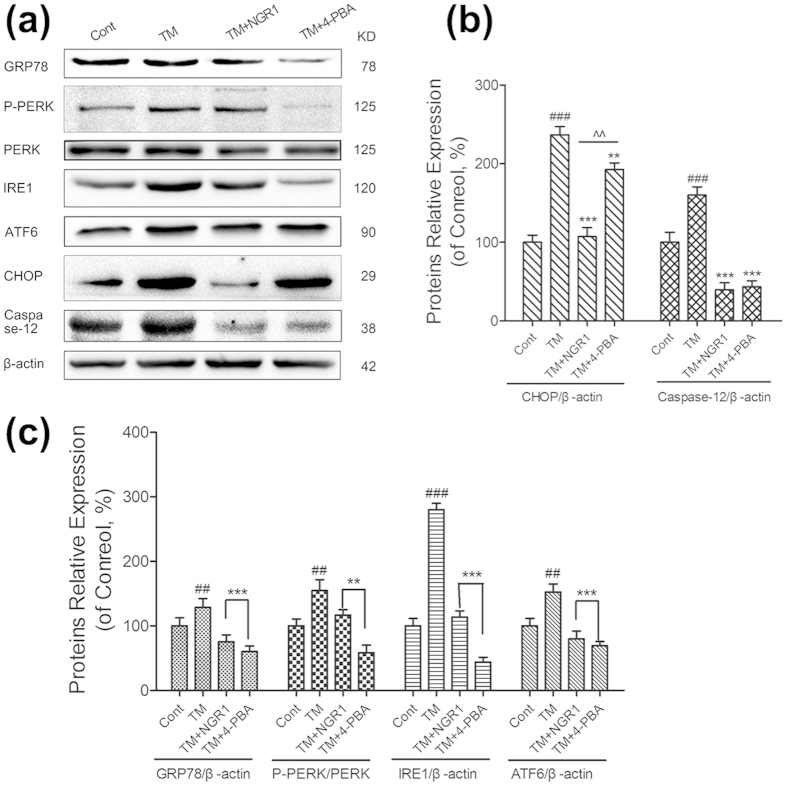
NGR1 suppressed TM-induced overexpression of ERS proteins. (**a**) The expression levels of ERS protein and the apoptosis proteins were detected using an immunoblotting analysis; (**b**,**c**) the relative protein expression of GRP78, IRE1, ATF6, CHOP, and Caspase-12 to β-actin, and P-PERK to PERK were expressed in the bar graphs. The results are expressed as the mean ± SD from three independent. ^##^*P* < 0.01 versus the control group, ^###^*P* < 0.001 versus the control group, **P < 0.01 versus the TM group, ***P < 0.001 versus the TM group, ^^^^*P* < 0.01 versus the TM + NGR1 group.

To address how NGR1 improved myocardial function on TM-impaired myocardium, we used westernblotting with the total soluble protein of the Langendorff-perfused rat hearts. As shown in Fig. 6c,d, TM treatment alone caused a significant upregulation in the protein expression levels of GRP78, ATF6, and IRE1 and in the phosphorylation of PERK and eIf2α compared with the control (*P* < 0.05 or *P* < 0.01), whereas 4-PBA alone significantly suppressed this protein expression. However, as shown in Fig. 7, both NGR1 and 4-PBA could significantly decrease the TM-induced overexpression of ERS proteins (GRP78 from 128.4 to 75.5, 60.1 of the control group, ATF6 from 152.5 to 79.7 and 69.1, PERK phosphorylation from 154.7 to 116.5 and 58.0, IRE1 from 279.8 to 113.7 and 43.8 of the control group, respectively,%), and 4-PBA showed better effects in suppressing phosphorylation of PERK and expression of IRE1 (Fig. 7a–c, *P* < 0.01 or *P* < 0.001). Interestingly, pharmacological intervention with NGR1 (20 μM) was more effective at decreasing the overexpression of CHOP (from 236.9 to 107.2) than 4-PBA (to 192.8) (Fig. 7a,b *P* < 0.01). Consistent with the results presented in Fig. 6, the data indicated that NGR1 inhibited TM-induced myocardial injury in both H9c2 cardiomyocytes and Langendorff-perfused rat hearts, which confirmed the involvement of ERS and all three UPR pathways in the NGR1 cardioprotection of I/R injury.

## Discussion

**Figure 8:**
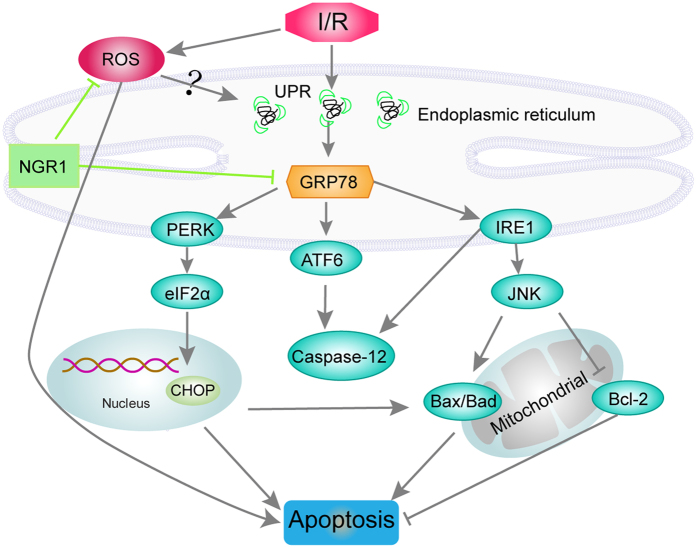
Summary scheme of the mechanisms underlying NGR1’s inhibition on I/R-induced ERS and apoptosis. I/R treatment induce the overexpression of GRP78 and accumulation of reactive oxygen species. The activation of GRP78 is able to induce a cascade of events that include the upregulation of ERS sensors ATF6, IRE1, and phosphorylation of PERK, resulting in the induction of apoptosis through CHOP, Caspase-12, and JNK-dependent pathways. NGR1 attenuate the up-regulated GRP78 expressions and inhibit the activation of ERS sensors, thus blocking the signalling pathways of ERS and their associated apoptosis. The inhibition of oxidative stress is relied on NGR1’s effects of scavenging free radical, abatementing the lipidperoxidament of cell membrane, and increasing the activity of antioxidase.

Cardiovascular diseases are currently the leading cause of morbidity and mortality worldwide, with over 7 million deaths per year^23^. Myocardium I/R injury leads to massive death of cardiomyocytes and plays a key role in the development of coronary heart diseases^4^,^24^. In the present study, we identified that NGR1 provides superior cardioprotective effects in inhibiting I/R injuries by reducing cardiac dysfunction, inhibiting myocardial apoptosis and improving contractile recovery both in isolated rat hearts and in H9c2 cardiomyocytes. What’s more, our results illustrated that the inhibition of oxidative stress- and ERS- associated apoptosis are involved in NGR1’s cardioprotection against I/R injury. We demonstrate that NGR1 is capable of scavenging free radical, abatementing the lipidperoxidament, and increasing the activity of antioxidase, thus suppressing oxidative stress. Besides, NGR1 ameliorates the I/R-induced death of cardiomyocytes and delays the onset of ERS by inhibiting the overexpression of GRP78 and ERS sensors PERK, ATF6, and IRE1 (Fig. 8). NGR1 can also protect the myocardium against TM-induced myocardial injury both *ex vivo* and *in vitro*, which further validates the involvement of ERS and three UPR pathways in the protective effects of NGR1.

Cell death during I/R is an active and multifactor process^25^,^26^. Recently, the role of the ERS in I/R injury has gained significant attention because ATP depletion, abnormal oxidative status and disrupted calcium homeostasis during cardiac I/R injury can cause the accumulation of misfolded proteins in the ER lumen^27^,^28^, which is known to trigger the unfolded protein response (UPR) and ERS^15^. ERS induces two major protective responses: attenuation of protein synthesis and an increase in the expression of genes that encode chaperones to facilitate the protein folding in the ER^29^,^30^. Three major signalling pathways are involved in ERS response: i) the RNA-dependent protein kinase-like ER kinase (PERK), which regulates cellular protein synthesis and limits additional influx of proteins into the lumen of the stressed ER^31^; ii) the ER transmembrane kinase or the inositol-requiring enzyme-1α (IRE1), a Ser/Thr kinase with an endonuclease domain that can remove 26 nucleotides from the mRNA of x-box binding protein 1 (XBP-1), which results in the translation of stable XBP-1 transcription factor to promote the ERS gene program^32^; and iii) the type II transmembrane protein or the activating transcription factor-6 (ATF-6), which can facilitate the ER folding capacity through induction of chaperone expression^33^. Although the ERS response is initially directed towards cellular adaptation to alleviate the unfolded protein load, prolonged ERS is associated with the activation of apoptosis^14^.

The importance of these pathways has been recognized, although few studies have been performed on their functional significance in the impaired heart, and only indirect evidence is available to suggest that I/R in the heart induces ERS^34^. In this regard, the central aim of the present study was to investigate whether and how ERS is involved in the protective effects of NGR1 during I/R. We established a myocardial ischemia/reperfusion (I/R) model in isolated rat hearts and a hypoxia/reoxygenation (H/R) model in H9c2 cardiomyocytes to mimic I/R injuries *ex vivo* and *in vitro*. 4-PBA was employed as positive control. Among the known chemical chaperones, 4-PBA has a high *in vivo* safety profile and has already been proved to have beneficial effects on several animal models of I/R injury by suppressing ERS and associated apoptosis, thus is known as an ER inhibitor^35^,^36^. We observed that NGR1 preconditioning ameliorated I/R-induced heart dysfunction in the Langendorff-perfused rat hearts and showed better and faster effects when compared with the 4-PBA positive control group (Fig. 4a,b, *P* < 0.05). Consistently, the *in vitro* results indicated that NGR1 (20 μM) treatment showed better cardioprotective effects than 4-PBA in H9c2 cardiomyocytes (Fig. 6b, *P* < 0.01).

Increased plasma myocardial enzyme activities are characteristic of myocardium I/R injury^8^,^37^. The generation of ROS was reported to damage the sarcoplasmic reticulum of heart, induces contractile dysfunction and Ca^2+^ release by modifying the structure and function of cardiac proteins^38^. Thus, antioxidant therapy can be effective in preventing oxidative stress-induced cell injury during I/R. Compared with the control, H/R treatment increased the extracellular LDH levels and the intracellular ROS levels in H9c2 cells (Fig. 2d, P < 0.01) and caused significant decreases in the activities of SOD, CAT, GSH-Px and increases in MDA and CK production in the Langendorff rat hearts (Fig. 4c, *P* < 0.01). However, these changes were effectively improved by NGR1 preconditioning in a dose-dependent manner, which indicates that reperfusion of the affected tissues triggers oxidative stress, and that NGR1 protects the myocardium from the oxidative stress and oxidative damage by mediating the antioxidant enzyme activities. We also examined the index of oxidative stress in the TM treatment groups, and no significant difference was found between the TM-impaired group and the control group (Supplementary Figure 1, *P* > 0.05).

It was first reported in 2000 that treatment with calcium ionophores, a sarcoplasmic/ER-calcium ATPase (SERCA) pump inhibitor (thapsigargin), or an inhibitor of N-linked glycosylation (tunicamycin), could initiate a form of apoptosis referred to as ERS-mediated apoptosis^39^. It has been well established that C/EBP homologous protein (CHOP) is a critical important pro-apoptotic transcription factor during ER-initiated apoptosis^40^,^41^, which can mediate transcriptional induction of BIM, a pro-apoptotic BH3-only protein while inhibiting Bcl-2, an anti-apoptotic protein. CHOP is downstream of the PERK-eIF2α-ATF4 pathway and the ATF6 pathway in UPR^42^. The activation of PERK enhances translation of ATF4, which subsequently induces the expression of CHOP, thus actively promoting apoptosis *in vivo* and *in vitro*. In addition, the cleaved ATF6 binds to ERSE in the CHOP gene to induce its transcriptional activation^43^. Further, phosphorylated JNK^44^ and activated Caspase-12^28^ are also involved in this process. The kinase domain of IRE1 was reported to activate c-Jun N-terminal kinase (JNK) by interacting with TNF receptor-associated factor 2 (TRAF2) and apoptosis signal-regulating kinase 1 (ASK1) in neuronal and pancreatic tumour cell lines^30^. Caspase-12 is a member of the interleukin-1b converting enzyme (ICE) subfamily of caspases, which is specific to the apoptosis mediated by ERS and is not proteolytically activated by other death stimuli^45^. The IRE1/TRAF2 complex also contributes to apoptosis through Caspase-12 released from the ER and the ensuing cell death^46^.

Our data showed that I/R significantly increased the relative protein levels of the ERS-responsive marker GRP78, the ERS sensors ATF6,PERK, eIf2α and IRE1, as well as the downstream apoptosis proteins, including CHOP, Caspase-12, P-JNK, BAX, and BAD, decreased the level of Bcl-2, which indicated the activation of ERS and its associated apoptosis in I/R (Figs 4e and 5, *P* < 0.05, *P* < 0.01, or *P* < 0.001). However, this I/R-induced processing was significantly suppressed by NGR1, *ex vivo* and *in vitro*. Compared with the I/R group, NGR1 treatment significantly increased myocardium Bcl-2 protein expression and decreased the protein expression of ERS-responsive proteins GRP78, P-PERK, ATF6, IRE1and apoptosis proteins CHOP, Caspase-12, etc, (Figs 4e and 5, *P* < 0.05, *P* < 0.01, or *P* < 0.001). In particular, P-PERK, and P-eIf2α were largely inhibited by NGR1 (20 μM) to 42%, 50% of that in I/R group in isolated heart tissue (Fig. 5a, *P* < 0.01 or *P* < 0.001), and 41%, 47% of that in the H/R group in H9c2 cells (Fig. 5b, *P* < 0.01 or *P* < 0.001), which indicated the direct inhibitory effects of NGR1 on ERS, especially the GRP78- PERK/eIf2α-associated pathways.

Besides, in the TM-impaired myocardium, the myocardial injury induced by TM was significantly attenuated by NGR1 treatment both in Langendorff-perfused rat hearts (Fig. 6a, *P* < 0.05) and in H9c2 cardiomyocytes (Fig. 6b, *P* < 0.01, or *P* < 0.001), which confirmed the involvement of ERS and all three UPR pathways in the NGR1 cardioprotection of I/R injury. TM initiated the activation of ERS by inhibiting the N-linked glycosylation of nascent proteins and leading to accumulation of misfolded and unfolded proteins. Since there was no oxidative stress reactions observed in TM-impaired myocardium (Supplementary Figure 1, *P* > 0.05), it revealed us that NGR1 is capable of acting directly on ERS without going through the oxidative stress pathways. Consistantly, NGR1 (20 μM) treatment showed better cardioprotective effects than 4-PBA on H/R-induced cell death in the H9c2 cardiomyocytes but did no better on improving the TM-induced cell death(Figs 2d and 4c, *P* < 0.01). Moreover, NGR1 is better on suppressing the TM-induced overexpression of an apoptosis protein (CHOP) but was not more effective at inhibiting ERS proteins (GRP78, PERK, IRE1, or eIf2α) compared with 4-PBA.

The ER is a highly dynamic organelle that exerts a major role in coordinating signalling pathways that ensure cell adaptation, cellular resilience, and survival^47^. Evidence for a role of ERS-mediated cell death in a variety of diseases make this process an attractive target for therapy^48^,^49^. Our results revealed the significant protective effects of NGR1 against I/R injuries, both *ex vivo* and *in vitro*, and illustrated that the cardioprotective effects of NGR1 were mediated partly by the suppression of oxidative stress- and ERS- associated apoptosis. However, the overall mechanisms underlying the cardioprotective effects of NGR1 and its association with ERS require further investigations.

## Materials and Methods

### Materials

Notoginsenoside R1 (NGR1, CID: 441934, molecular weight = 933.15; purity > 98%) was supplied by Shanghai Winherb Medical S&T Development (Shanghai, China). Tunicamycin (TM) from Streptomyces was purchased from Sigma (St. Louis, MO, USA), and the 4-phenylbutyric acid (4-PBA, CAS.NO. 1821-12-1) was purchased from Sinopharm Chemical Reagent Co., Ltd (Beijing, China). All cell culture materials, Dulbecco’s modified Eagle’s medium (DMEM), foetal bovine serum (FBS), and penicillin/streptomycin were purchased from Gibco (NY, USA). The kits for determining the malondialdehyde (MDA) content and theactivity of creatine kinase (CK), catalase (CAT), lactate dehydrogenase (LDH), glutathione peroxidase (GSH-Px), and superoxide dismutase (SOD) were obtained from Jiancheng Bioengineering Institute (Nanjing, China). Primary antibodies against JNK, P-JNK, CHOP, GRP78, ATF6, P-PERK, PERK, IRE1, eIf2α, P-eIf2α, Caspase-12, Bcl-2, BAX, BAD and β-actin were obtained from Santa Cruz Biotechnology (CA, USA).

### Cell Culture and hypoxia-reoxygenation (H/R) modelling

Rat embryonic cardiomyoblast- derived H9c2 cardiomyocytes were obtained from the Cell Bank of the Chinese Academy of Sciences (Shanghai, China) and cultured in high glucose DMEM, supplemented with 10% foetal bovine serum, 1% penicillin/streptomycin. For all of the experiments, the cells were plated at an appropriate density and were grown in a humidified incubator containing 5% CO_2_ at 37 °C for at least 24 h to reach 70–80% confluence before experimentation.

The H9c2 cells were pretreated with indicated concentrations (2.5, 5, 10, 20, 40 or 80 μM) of NGR1 or 4-PBA (3 mM) for 24 hours and then exposed to H/R or TM. The H/R model was built using a modified process^50^. Briefly, the H9c2 cardiomyocytes were incubated at 37 °C in an anaerobic glove box (Coy Laboratory, USA), where normal air was replaced by a combination of 5% CO_2_, 5% H_2_, and 90% N_2_, with the high glucose DMEM medium replaced by no-glucose DMEM to mimic ischemia. The cells were cultured under hypoxia for 6 h, and then, removed to the regular incubator with the medium replaced by high glucose medium and were maintained for 12 h to mimic reperfusion. When employing TM to cause cell damage, H9c2 cardiomyocytes were incubated with TM (3 μM) for 6 h. The corresponding control cells were incubated under normoxic conditions for equivalent durations with high glucose DMEM without FBS.

### Cell viability and LDH concentration

The cell viability of the H9c2 cardiomyocytes was determined using a MTT assay. Cells cultured in 96-well plates (1 × 10^4^ cells/well) were incubated with MTT solution (1 mg/ml final concentration) at 37 °C for 4 h after the various treatments. The formazan crystals were dissolved with dimethyl sulfoxide (DMSO, 100 ml/well), and the absorbance was detected at 570 nm on a microplate reader (SpectraFluor, Tecan, Sunrise, Austria). Cell viability was expressed as the percentage of MTT reduction compared with the control conditions.

Cell death was evaluated by LDH leakage. The medium of the H9c2 cardiomyocytes cultured in 6-well plates was collected to measure the LDH release using an LDH assay kit according to the manufacturer’s instructions.

### Intracellular ROS Production

Cells were harvested, washed with 1 × washing buffer, and then incubated with 5-(and-6)-carboxy-2′, 7′-dichlorodihydrofluorescein diacetate (carboxy-H2DCFDA) at a final concentration of 25 μM in the dark at 37 °C for 30 min. The fluorescence was analyzed using a FACSCalibur flow cytometer (BD, Biosciences, CA, USA).

### Mitochondrial transmembrane potential (ΔΨm)

The changes in mitochondrial transmembrane potential were detected by 5,5′, 6,6′-tetrachloro- 1,1′, 3,3′-tetraethylbenzimidazolyl-carbocyanine iodide (JC-1) as previously reported^51^. H9c2 cardiomyocytes (1 × 10^5^ cells/well) were cultured in 96-well plates. After precondition with NGR1 for 24 h and H/R, the cells were incubated with JC-1 at a final concentration of 2 μM in the dark at 37 °C for 15 min. Images of the cells labelled with JC-1 were observed under a high-content imaging system Image Xpress Micro (Molecular Devices, USA).

### Hoechst 33342 and Propidium Iodide (PI) Double Staining

The H9c2 cardiomyocytes were washed twice with PBS and incubated with 10 mg/ml of Hoechst 33342 (Sigma, USA) dye for 15 min, and 100 mg/ml of PI (Sigma, USA) was then added. The stained nuclei were immediately observed using Image Xpress Micro (Molecular Devices, USA).

### Terminal Deoxynucleotidyl Transferase-mediated dUTP Nick End Labelling (TUNEL) Staining

Apoptotic H9c2 cardiomyocytes were visualized using TUNEL staining according to the manufacturer’s instructions^52^. H9c2 cardiomyocytes were cultured on cover slips. After the treatment, the cells were fixed with 4% neutral buffered formalin solution for 30 min. After twice washes with PBS, images were captured using a fluorescence microscope (Leica, Germany), and the apoptotic cells were counted with at least 100 cells from four randomly selected fields in each group.

### Animals and treatments

Male Sprague-Dawley rats, weighing 200–220 g (8 weeks of age), were purchased from Beijing Vital River Laboratory Animal Technology Co., Ltd., Beijing, China. The animals were housed under standard laboratory conditions (temperature of 22 ± 2 °C, humidity of 60% ± 10%, and light from 6 a.m. to 6 p.m.), given standard rodent chow, and allowed free access to water. All of the procedures were performed in accordance with the guidelines of the Experimental Laboratory Animal Committee of Chinese Academy of Medical Sciences and Peking Union Medical College and the principles and guidelines of the National Institutes of Health Guide for the Care and Use of Laboratory Animals.

The Langendorff operation was performed as a previously described procedure^53^,^54^. Briefly, rats were anesthetized with urethane (20%), and their hearts were rapidly removed according to University of Chicago Institutional Animal Care and Use Committee-approved protocols. The aorta was mounted on a Langendorff perfusion apparatus with oxygenated Krebs-Henseleit (KH) buffer (11 mM glucose, 118 mM NaCl, 25 mM NaHCO_3_, 4.8 mM KCl, 1.2 mMKH_2_PO_4_, 1.2 mM CaCl_2_, 1.7 mM MgSO_4_, 0.7 mM Na pyruvate, saturated with 95% O_2_–5% CO_2_, pH 7.4 at 37 °C), and the heart was paced at a cycle length of 200 ms (300 bpm). LV pressure was measured using a water-filled wrap balloon connected to a pressure transducer (AD Instruments, Sydney, NSW, Australia). All hearts were equilibrated with KH buffer for 15 min before the application of experimental protocols. NGR1 (dissolved in perfusate, 5 μM, 10 μM, or 20 μM) or 4-PBA (dissolved in hot KH buffer, 3 mM) was added to the perfusate to act on the hearts for 15 min. Then, the hearts were subjected to 40 min of global ischemia followed by 60 min of reperfusion. Tunicamycin (TM, 3 μM) was added to the perfusate to act on the hearts for 15 min and was followed by 80 min of regular KH buffer perfusion.

The rats were randomly assigned to 10 groups of ten rats each:

- (1) Control group, perfusion at 37 °C for 115 min;
- (2) I/R group, I/R at 37 °C with DMSO added to the perfusate for 15 min before the I/R;
- (3) (4) (5) NGR1 + I/R groups, similar to the I/R group, but DMSO is replaced by NGR1 (5 μM, 10 μM, or 20 μM) dissolved in perfusate;
- (6) NGR1 group, 20 μM NGR1 was added to perfusate to act for 15 min, perfusion with pure perfusate for another 100 min;
- (7) 4-PBA + I/R group, similar to NGR1 + I/R groups with NGR1 replaced by 4-PBA (3 mM);
- (8) TM group, similar to the NGR1 group, but with NGR1 replaced by TM (3 μM);
- (9) NGR1 + TM group, 20 μM NRG1 acted on heart for 15 min followed by 3 μM TM for 15 min;
- (10) 4-PBA + TM group, similar to group (9), but with NGR1 replaced by 3 mM 4-PBA.

### Ethics Statement

All procedures in this study were performed following the regulations of the Chinese Guide for the Care and Use of Laboratory Animals published by the United States National Institutes of Health Publication No. 85–23, revised 1996, and approved by the Experimental Laboratory Animal Committee of Chinese Academy of Medical Sciences and Peking Union Medical College. All sacrifices were performed under pentobarbitone anesthesia, and every effort was made to minimize animal suffering.

### Heart Histopathological Examination

The Langendorff-perfused rat hearts were fixed with 4% paraformaldehyde for more than 48 hours. Then, the left ventricles of the hearts were dissected and embedded in paraffin blocks, sectioned, stained with hematoxylin and eosin (HE), and examined under a light microscope (CKX41, 170 Olympus, Tokyo, Japan) by a pathologist who was blinded to the groups under study.

### Antioxidant indices in the hearts

The heart tissues after the Langendorff processing were homogenized (10% w/v) with phosphate buffer (pH 7.4) and centrifuged at 5000 rpm for 15 min. The supernatant was used to estimatethe reactive oxygen species levels by measuring the tissue content of MDA and GSH-Px and the activities of SOD, CAT, and CK^55^,^56^. Detailed manipulation processes were performed according to the manufacturer’s instructions.

### Western blotting

Total soluble protein was extracted from the left ventricle of the hearts using extraction buffer supplemented with 1 mM PMSF. Equal amounts of protein samples from the different groups were separated using SDS-PAGE and transferred onto nitrocellulose membranes^57^. Immunoblotting analysis was performed by incubating the membrane overnight with corresponding primary antibodies. Then, the membranes were incubated with secondary antibody conjugated with horseradish peroxidase at a 1:1000 dilution. The intensities of bands were determined using a densitometer (Molecular Devices, CA, USA) and the AlphaEaseFC™ software. β-actin was used as an internal standard.

### Statistical analysis

All experiments were repeated three times. The results are presented as the mean ± S.D. The differences between the groups were analyzed using one-way analysis of variance followed by Student-Newman-Keuls post hoc test. *P* < 0.05 was considered statistically significant.

## Additional Information

**How to cite this article**: Yu, Y. *et al*. Cardioprotective effects of Notoginsenoside R1 against ischemia/reperfusion injuries by regulating oxidative stress- and endoplasmic reticulum stress-related signaling pathways. *Sci. Rep.* **6**, 21730; doi: 10.1038/srep21730 (2016).

## Supplementary Material

Supplementary Information
