# Supplementary material for: Cardioprotective effects of Notoginsenoside R1 against ischemia/reperfusion injuries by regulating oxidative stress- and endoplasmic reticulum stress- related signaling pathways
Source: Sci Rep. 2016 Feb 18;6:21730. doi: 10.1038/srep21730 (PMC4757886; doi:10.1038/srep21730)
Supplement: Supplementary Information [file srep21730-s1.pdf]

Cardioprotective effects of Notoginsenoside R1 against ischemia/reperfusion injuries by regulating oxidative stress- and endoplasmic reticulum stress- related signaling pathways

Yingli Yu[1,2,3], Guibo Sun[1,2,3]*, Yun Luo[1,2,3], Min Wang[1,2,3], Rongchang Chen[1,2,3], Jingyi Zhang[1,2,3], Qidi Ai[1,2,3], Na Xing[4], Xiaobo Sun[1,2,3]*

1 Beijing Key Laboratory of Innovative Drug Discovery of Traditional Chinese Medicine (Natural Medicine) and Translational Medicine, Institute of Medicinal Plant Development, Peking Union Medical College and Chinese Academy of Medical Sciences, Beijing, 100193, China

2 Key Laboratory of Bioactive Substances and Resource Utilization of Chinese Herbal Medicine, Ministry of Education

3 Zhongguancun Open Laboratory of the Research and Development of Natural Medicine and Health Products

4 Key Laboratory of Chinese Materia Medica, Heilongjiang University of Chinese Medicine, Harbin, 150040, China.

*Correspondence authors. Address: Institute of Medicinal Plant Development, Chinese Academy of Medical Sciences & Peking Union Medical College, No. 151, Malianwa North Road, Haidian District, Beijing 100193, PR China. Tel: +86-010-57833013; Fax: +86-010-57833013. E-mail addresses: sunguibo@126.com (Guibo Sun), sun_xiaobo163@163.com (Xiaobo Sun).

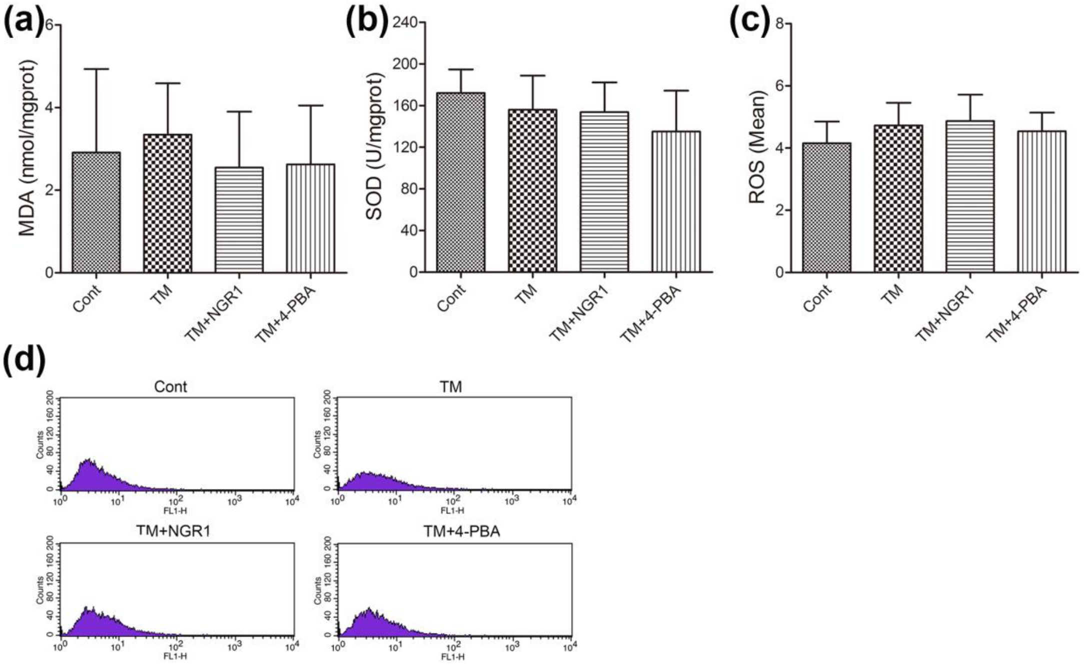

Supplementary Figure 1. Effects of NGR1 on redox state and intracellular ROS accumulation in TM-impaired isolated rat hearts and H9c2 cardiomyocytes. The SOD activity (a) and the MDA production (b) of the isolated heart were examined; Intracellular ROS levels were evaluated using a FACSCalibur flow cytometer(d) and showed in bar diagram (c).
